# Investigating the Clinical Characteristics and *PITX3*Mutations of a Large Chinese Family with Anterior Segment Mesenchymal Dysgenesis and Congenital Posterior Polar Cataract

**DOI:** 10.1155/2023/1397107

**Published:** 2023-04-24

**Authors:** Hui Dang, Min Peng, Weiyue Gu, Gang Ding, Yuqin Sun, Zhongkai Hao, Ning Wei, Xu Wang, Chenming Zhang, Aijun Deng

**Affiliations:** ^1^Department of Ophthalmology, Jinan Second People's Hospital, Jinan 250200, China; ^2^Zhigene Translational Medicine Research Center Co. Ltd., Beijing 100176, China; ^3^Department of Ophthalmology, Affiliated Hospital of Weifang Medical University, Weifang 261000, China

## Abstract

**Objective:**

To investigate the clinical characteristics and pathogenic genetic mutations of a Chinese family with anterior segment mesenchymal dysgenesis and congenital posterior polar cataract.

**Methods:**

Through family investigation, the family members were examined via slit lamp anterior segment imaging and screened for eye and other diseases by eye B-ultrasound. Genetic test was performed on the blood samples of the fourth family generation (23 people) via whole exome sequencing (trio-WES) and Sanger sequencing.

**Results:**

Among the 36 members in four family generations, there were 11 living cases with different degrees of ocular abnormalities, such as cataracts, leukoplakia, and small cornea. All patients who received the genetic test had the heterozygous frameshift mutation c.640_656dup (p.G220Pfs*∗*95) on exon 4 of the PITX3 gene. This mutation was cosegregated with the clinical phenotypes in the family and thus might be one of the genetic factors that cause the corresponding ocular abnormalities in this family.

**Conclusion:**

The congenital posterior polar cataract with or without anterior interstitial dysplasia (ASMD) of this family was inherited in an autosomal dominant manner, and the frameshift mutation (c.640_656dup) in the PITX3 gene was the cause of ocular abnormalities observed in this family. This study is of great significance for guiding prenatal diagnosis and disease treatment.

## 1. Introduction

Anterior segment mesenchymal dysgenesis (ASMD) refers to a series of complex abnormalities of the anterior eye segment, which are usually manifested as corneal opacity, Peters' abnormality, abnormal position of the posterior embryonic ring, Schwalbe's line, iris hypoplasia, corneal flexion or polycornea, adhesions between the iris and the cornea, and early continuous development of cataracts [1, 2]. Congenital cataract refers to lens opacity that occurs during birth or in the first ten years of life. It is one of the main causes of blindness in children. According to the clinical manifestations and the characteristics of the lens opacity distribution, congenital cataract can be classified into lamellar, nuclear, cortical, dusty, posterior polarity, blue dot, and anterior polar cataracts. The main causes of congenital cataract are genetic factors, embryonic maternal infection, vitamin deficiency, and environmental factors, among which genetic mutations account for more than 30% of congenital cataracts [3]. Congenital cataracts are inherited via autosomal dominant inheritance, autosomal recessive inheritance, and X-linked inheritance, and the autosomal dominant congenital cataract (ADCC) is the most common type. Congenital cataract can occur as an isolated defect or co-occur with other congenital abnormalities in the eyes or other systems, such as anterior segment mesenchymal dysgenesis (ASMD), glaucoma, small cornea or aniridia, Lowe syndrome, Nance Horan syndrome, and Wolfram syndrome.

Among the currently identified proteins encoded by genes known to cause congenital cataracts, about half are proteins with enriched expression in the lens (e.g., crystallins), a quarter is membrane proteins, and the rest are cytoskeleton proteins and transcription factors. Cataracts have obvious phenotypes and genetic heterogeneity. The mutations in genes that are critical to the development and maintenance of the lens structure (such as lensin, connexin, and aquaporin) are usually associated with isolated congenital cataracts, and the transcription factors *PAX6*, *FOXE3*, *EYA1*, *MAF*, and *PITX3* have also been reported in congenital cataracts with ASDM [4]. Transcription factors play a key role in the development of eyes and other embryonic tissues, and the mutations in these genes often lead to serious developmental defects. *PITX3* encodes a highly conserved PITX family homeobox transcription factor, which regulates lens development and substantia nigra dopaminergic neurons [5]. It has been reported that most of the cataracts caused by *PITX3* mutation are posterior polar cataracts, sometimes accompanied with ASMD. These diseases are usually curable, such as cataracts and leukoplakia, but they can also lead to blinding eye diseases. In this study, we analyzed the clinical phenotypes and genetic characteristics of a four-generation family with ASMD in Shandong, China. Our results provided more evidence for the early screening and diagnosis of this type of disease.

## 2. Materials and Methods

### 2.1. Family Data Survey and Sample Collection

**Figure 1:**
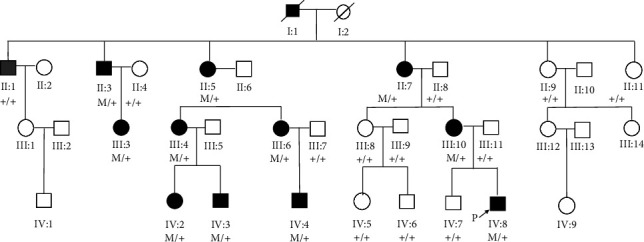
Pedigrees of the Chinese family with congenital posterior polar cataract via autosomal dominant inheritance. Square: male; round: female; black symbols: patient; white symbols: healthy individual; slash: passed away; arrow: the proband; M: variant; +: wild type.

A Chinese Han family admitted to the Jinan Eye Hospital in Shandong, China, was analyzed in this study. The family had 4 generations, in which the data from 36 members can be retrospectively investigated (Figure 1), including 12 people with cataracts (5 males and 7 females). 23 family members, including the proband, were enrolled in this study, and there were 11 affected individuals and 12 unaffected individuals. After all the cases signed the informed consent, their medical histories were collected, and participants underwent detailed eye examinations, including visual acuity, slit lamp examination, fundoscopy, and intraocular pressure measurement. 5∼10 ml of peripheral blood samples was collected into EDTA-coated anticoagulation tubes. Gentra Puregene blood kit (Qiagen, Germany) was used to extract genomic DNA from the blood samples, and UV spectrophotometry (ActGene Inc, Taipei, Taiwan) was used to examine the quantity and purity of the extracted DNA, which were then store at −20°C for later use. All experiments and sampling procedures were approved by the Ethical Review Committee of Jinan Eye Hospital (No. JNEYE20230608). All procedures were conducted in accordance with the 1964 Helsinki Declaration and subsequent amendments or similar ethical standards.

### 2.2. Whole-Exome Sequencing and Candidate Gene Analysis

The patient DNA samples (patient: IV: 8, III: 10, control: IV: 7, and III: 11) were sent to the Beijing Full Spectrum Medical Laboratory for proband whole-exome sequencing (WES). The NimbleGen whole-exon capture probe was liquid hybridized with the gDNA library sequence to enrich the DNA fragments in the target area, and the whole exon library was constructed. Illumina Hiseq sequencer was used to perform high-throughput sequencing (PE150), and the sequence coverage of the target sequence was not less than 99%. After quality control, the sequencing data were mapped to the Ensemble reference genome GRCh37/hg19 using Burrows–Wheeler Aligner (BWA) software, and the SNP and Indel were analyzed using GATK software. Then, the high-quality and reliable mutations were obtained by filtering the detected SNP and Indel according to the sequencing depth and mutation quality. The self-developed variant annotation software was used to perform association annotations on the detected high-quality variants in major databases (such as dbSNP, thousand genomes, ExAC, ESP, OMIM, HGMD, and ClinVar). With the help of protein structure prediction software such as Provean, SIFT, Polyphen2-HVAR, Polyphen2-HDIV, M-Cap, REVEL, MutationTaster, and cleavage site prediction software such as MaxEntScan, we analyzed the effects of mutations and screened out the mutations that might have negative effects on the protein structure. According to the 2015 ACMG diagnostic guidelines of the United States and the 2018 “Clinical Single Gene Genetic Disease Gene Test Reporting Specification,” we performed pathogenicity analysis on the mutation loci. The mutation found by WES was verified by Sanger sequencing. The target DNA regions of all patients were PCR amplified and sent for Sanger sequencing by ABI3730 sequencer. Then, the family transmission analysis was performed.

## 3. Results

### 3.1. Clinical Phenotypes

#### 3.1.1. Eye

**Figure 2:**
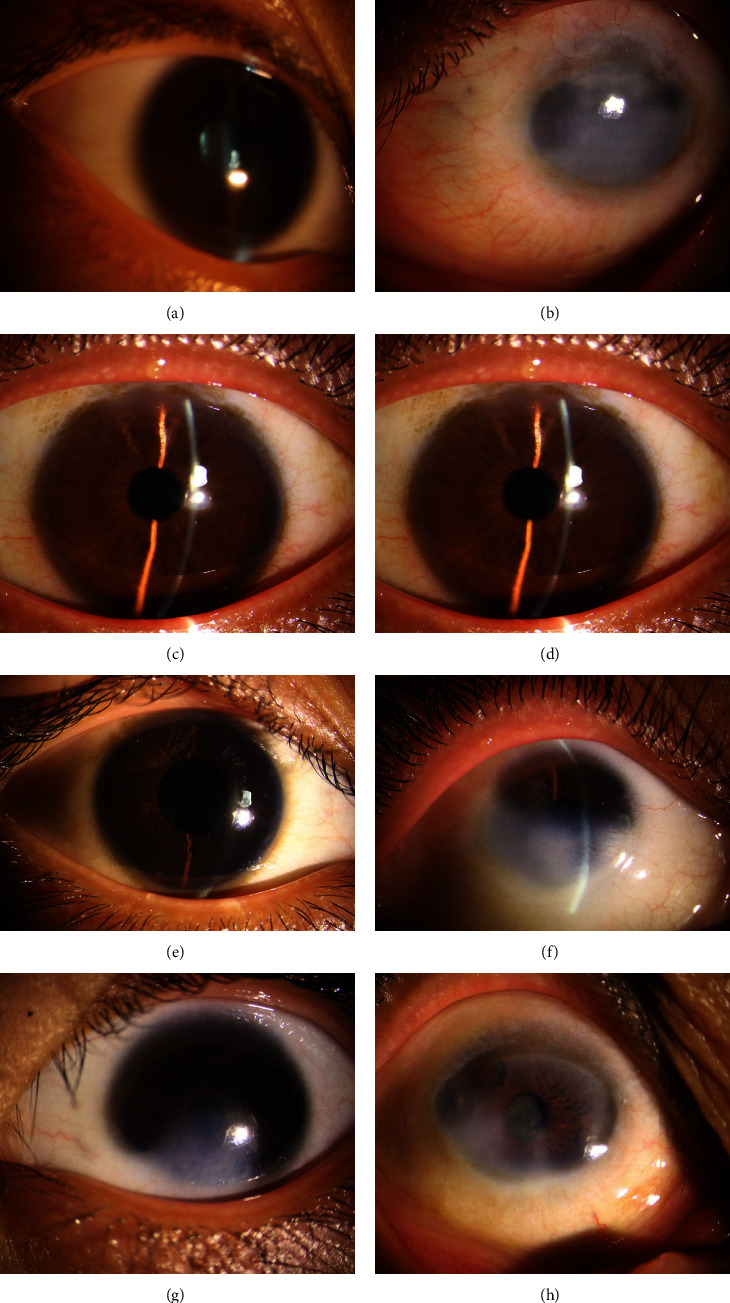
Slit lamp examination of the patients. (a) Patient IV: 4: subcapsular punctate opacities at the posterior pole of the lens; (b) patient II: 3: corneal leukoplakia, about 8 mm in diameter, the inner eye structure is not visible; (c) patient III: 4: left eye received cataract surgery, upper iris injury and partial depigementation can be observed; (d) patient III: 6: left eye received cataract surgery, the position of intraocular lens was normal; (e) patient IV: 2: right eye received cataract surgery, the position of intraocular lens was normal; (f) patient III: 3: congenital leukoplakia + congenital small cornea, no surgery; (g) patient IV: 3: congenital leukoplakia + congenital small cornea, no surgery; and (h) patient II: 7: congenital corneal leukoplakia + congenital small cornea + congenital cataract.

The family has 36 members (Figure 1) and 34 are currently alive, including 11 affected members (4 males and 7 females), ranging from 68 -days to 70 -years-old. Different individuals exhibited different phenotypes (Figure 2). Five cases showed congenital posterior polar cataracts (III: 4, III: 6, III: 10, IV: 2, IV: 4, the first 4 cases had undergone cataract extraction and intraocular lens implantation, best vision: greater than or equal to 10/20; IV: 4 did not undergo surgery, and best vision: 8/20); 3 cases showed congenital cornea leukoplakia + small cornea (III: 3, IV: 3, IV: 8, the latter two had undergone penetrating keratoplasty, IV: 3 had normal iris; the anterior iris adhesions were found in IV: 8 during the operation, the lens were transparent, and ASMD was considered; current vision of III: 3: 2/20; IV: 3 and IV: 8 had corneal graft rejection, and their current vision were HM); 3 cases showed congenital corneal leukoplakia + small cornea + congenital cataract (II: 3, II: 5, II: 7, of which II: 3 had undergone cataract extraction 30 years ago, without intraocular lens implantation; this case received penetrating keratoplasty at our hospital, anterior iris adhesion and lens loss were observed during the operation, and ASMD was considered) (Table 1). The visual acuity of II: 3 improved from HM (preoperative) to FC/20 cm (postoperative), and the visual acuity of II: 5 and II: 7 was both HM. All family members did not have marriage history with close relatives. The genetic characteristics of this family were as follows: each generation had individuals with cataracts diseases, but the phenotypes were different; the disease occurred in both males and females, and the inheritance was not related to gender; when the parents were healthy, the offspring were healthy, while when the offspring had the disease, at least one of the parents must have the disease; therefore, the inheritance mode was common chromosome dominant inheritance. The proband (IV: 8) came to our hospital at 68 -days-old with congenital leukoplakia + small cornea on both eyes. The mother of the proband (III: 10) had posterior polar cataract and received cataract extraction and intraocular lens implantation. Her current visual acuity is 8/20. All affected members have the same heterozygous frameshift mutation but exhibited different phenotypes.

#### 3.1.2. Whole Body

All 23 family members received physiological examination at the Neurology Department, and no one had Parkinson's disease.

### 3.2. Genetic Sequencing Results

**Figure 3:**
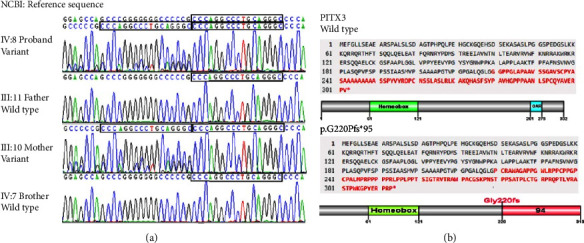
Sanger sequencing of *PITX3* gene mutation. (a) The *PITX3* gene mutation was not detected in healthy individuals in the family (reference transcript: NM_005029), and the heterozygous mutation c.640_656dup (p.G220Pfs*∗*95) was detected in the affected individuals, with 17 bp inserted at 657 bp position (as shown in the box). (b) The duplication of 17 bp fragment in the *PITX3* gene c.640_656dup (p.G220Pfs*∗*95) results in a frameshift starting from 220 amino acid position and a 313aa aberrant protein.

The whole-exome sequencing (WES) results showed that the *PITX3* gene in proband IV: 8 and his affected mother III: 10 had heterozygous frameshift mutation c.640_656dup (p.G220Pfs*∗*95) on exon 4; specifically, there was a 17 bp repeated sequence CCCAGGCCCTGCAGGGC inserted after the 656^th^ bp of the *PITX3* coding region, which resulted in a frameshift from the 220^th^ amino acid, forming an abnormal protein including 94 additional amino acids. Neither the father nor the older brother of the proband carried the mutation. Sanger sequencing was performed to verify the target sequence on 23 members in the family, and the mutation was confirmed to be cosegregated in the affected family members. All the 11 affected members carried the heterozygous mutation, while the remaining 13 unaffected members did not (Figures 1 and 3). We analyzed the mutation according to the American Society of Medical Genetics and Genome (ACMG) guidelines, and this mutation was a frameshift mutation and a loss-of-function mutation, resulting in possible loss of function in *PITX3* gene (PVS1); this mutation was not found in normal control population databases (ExAC, thousand genomes, DbSNP, etc.) (PM2). The evidence strength of this mutation was “PVS1 + PM2,” and so it was classified as a pathogenic mutation. The proband was heterozygous, which conformed to the pathogenesis of autosomal dominant (AD) disease, and the phenotypes and genotypes of the proband and his parents followed the cosegregation law. The *PITX3*gene-associated diseases are cataract 11 (CATARACT 11, MULTIPLE TYPES; CTRCT11; OMIM: 610623; AD, AR) and anterior segment hypoplasia type 1 (ANTERIOR SEGMENT DYSGENESIS 1; ASGD1; OMIM: 107250; AD), which are consistent with the phenotypes of posterior polar cataract, corneal opacity, anterior segment hypoplasia, and other phenotypes in this family. Since the clinical phenotypes matched, this mutation is the pathogenic mutation causing the corresponding eye abnormalities in the family.

## 4. Discussion

Transcription factors such as *PAX6*, *FOXE3*, *HSF4*, *MAF*, and *PITX3* are essential for eye development. The mutations in these genes can lead to serious eye defects and congenital cataracts. *PITX3* is located on chromosome 10q25 and has 4 exons. It is a member of the RIEG/PITX homeobox gene family and encodes a protein of 302 amino acids, which belongs to the paired homeobox transcription factor class. PITX3 protein has two different domains, the N-terminal homeobox domain and the C-terminal otp, aristalless, and rax (OAR) domain; these domains play an important role in DNA binding and transactivation activities [6]. *PITX3* is expressed in the developing lens, skeletal muscle, and substantia nigra dopaminergic neurons in the brain, playing a key role in the lens development during the formation of the vertebrate eyes. The mutation in *PITX3* is associated with abnormal development of the anterior eye, especially the lens and its polymorphism is related to Parkinson's disease, dementia, and neurological abnormalities [7, 8]. Mouse Pitx3 protein has 99% homology with human PITX3. It is essential for lens development during the early mouse embryonic development and is mainly expressed in the midbrain, tongue, sternum, spine, and other parts in later developmental stages [9]. The mouse *Pitx3* gene is located near the ak site of mouse chromosome 19. Consistently, the ak/ak homozygous mice did not have lens or eyelids, exhibiting microphthalmia, and the number of dopaminergic neurons was significantly reduced [10, 11]. The expression of *β*- and *γ*-crystallin was abnormal in the early developmental stage of Pitx3-deficient mice, indicating that Pitx3 has a unique function in the maintenance of epithelial cells and the initiation of fibrous differentiation [12]. Similarly, the knockdown of PITX3 gene in zebrafish can lead to abnormal development of retina and lens [13].

Unlike other transcription factors that cause ASMD, *PITX3* gene mutations can lead to solitary cataracts with or without ASMD (postpolar cataract is the main feature). The pathogenesis mechanisms include dominant and recessive cataracts, with or without other eye abnormalities. So far, 14 studies have reported 11 unique PITX3 mutations that can cause congenital cataracts [14–29], including congenital or early childhood cataracts, ASMD, corneal opacity, microphthalmia, microcornea, nystagmus, glaucoma, and other eye defects. Among these mutations, there is one missense mutation (c.38 G > A, p.Ser13Asn), one in-frame mutation (c.797_814del, p.S266_A271del), and the remaining 9 are all frameshift mutations (c.542delC, p.Pro181LeufsX127; c.573delC, p.Ser192AlafsX117; c.582delC, p.Ile194MetfsX115; c.608delC, p.Ala203GlyfsX106; c.640_656del, p.Ala214ArgfsX42; c.650delG, p.Gly217AlafsX91; c.669delC, p.Leu225TrpfsX84; c. 640_656dup17, p.Gly220ProfsX95; c.470−477dup, and p.Ala160ArgfsX152) (Table 2). Except the c.38 G > A (p.S13N) mutation, all the other mutation are located on exon 4. Moreover, only the p.S13N mutation is located upstream of the homeobox domain and has been reported twice; one was associated with complete cataracts and glaucoma [14], and one was associated with Peters' abnormality [26]. All the other mutations are located downstream of the homeobox domain and lead to abnormal expression or deletion of the C-terminal protein containing OAR domain; these mutations are associated with cataracts and other eye diseases with varying degrees, ranging from mild phenotypes such as postblastotoxin, to more serious phenotypes such as Peters' abnormalities, most of which are isolated posterior polar cataract or combined with other ocular abnormalities. Only one newly reported case of c.470_477dup was related to nuclear cataract [29]. Most of these mutations are dominant inheritance, and only three mutations, c.650delG, c.640_656del, and c.669delC, have been reported to have recessive inheritance. There were 4 homozygous patients from 3 close relatives, and they exhibited more serious ocular phenotypes (1/4 ASMD, 3/4 ophthalmia) than heterozygous family members and might be accompanied by neurological features (2/4 developmental delay); c.650delG homozygous mutation caused bilateral microphthalmia (with corneal opacity) and was related to severe developmental delay and nerve defects [18]; c.640_656del homozygous mutation caused severe microphthalmia, anterior segment dysplasia, and corneal sclerosis [23]; c.669delC homozygous mutation caused ASMD, bilateral congenital corneal sclerosis, and other deformities such as altered facial features [26]. In contrary, 650delG heterozygous mutation only led to progressive posterior polar cataracts [17, 19], c.640_656del heterozygous mutation caused congenital posterior subcapsular cataract [27], and c.669delC heterozygous mutation only led to congenital cataract and postblastotoxin [26]. These findings suggested that homozygous patients had a doubled dose of dominant mutation. c.582delC is the only newly reported mutation and it caused more severe phenotypes. In addition to congenital cataracts, this patient also showed bilateral microphthalmia, corneal opacity, developmental delay, and autism [26]. The neural system abnormity may be related to PITX3 mutation, but it is not yet certain whether other phenotypes (such as autism and facial features) are related to PITX3 mutation.

Since the first report by Semina et al., the 17 bp repeat insertion mutation c. 640_656dup (p.Gly220ProfsX95) has been identified as the most common mutation of the *PITX3* gene, accounting for more than half of the studied families (14/28) (Table 2). Electrophoretic migration analysis (EMSA) and luciferase reporter gene analysis showed that p.Gly220ProfsX95 mutation reduced the DNA binding and transactivation activities of PITX3 by changing the protein length [24, 30]. There were a total of 164 cataract patients in the 14 autosomal dominant postpolar cataract families with 17 bp repeat insertion mutations, while only 20 cases were accompanied by ASMD. Currently, there are 4 studies showing that *PITX3* gene is related to ASMD. One study is a molecular genetic study on 5 unrelated families with autosomal dominant postpolar cataract; a 17 bp insertion mutation was detected in 3 British families and 1 Chinese family, and only 4 patients from 2 British families showed ASMD and cataract [15, 16]. One study on Australian cataract patients found that all clinically affected family members had the same 17 bp insertion mutation, which can lead to treatable cataracts and minimal visual impairment and can also result in severe symptoms and functional blindness [20, 21]. One study on 5 Belgian ADCC and ASMD families found that 4 families were c.640_656dup heterozygous and 1 family had c.573delC mutation; they verified that these two variants had similar nuclear localizations and both reduced DNA binding and transactivation activities [24]. One study performed targeted sequencing to sequence 187 genes related to ocular development in 96 patients with ocular and ophthalmia, and they found that in 2 families, the c.640_656dup heterozygous mutation was cosegregated with eye abnormalities ranging from congenital cataracts to Peters' abnormality [26]. These studies indicate that the 17 bp repeat insertion mutation c. 640_656dup (p.Gly220ProfsX95) of the *PITX3* gene is the main gene mutation that causes ASMD; however, the number of ASMD cases is relatively small, and the ocular involvement of heterozygous patients is highly variable, suggesting that the penetrance of *PITX3* mutation is not complete, which may be due to the changes in the protein activity at different developmental stages caused by the stochasticity during development, the influence of gene modifications, and the intrauterine environment. In this study, we reported a 36-member family from East China with eye diseases caused by the 17 bp insertion mutation in the *PITX3* gene. The clinical phenotypes of this family included congenital cataracts, microphthalmia, and homogeneous corneal opacity, which is one of the characteristic phenotypes of anterior interstitial dysplasia. We performed peripheral blood whole-exon sequencing on a child patient (proband) in the family, as well as his parents and brother, and we found that the c.640_656dup heterozygous 17 bp insertion mutation on exon 4 of the *PITX3* gene was cosegregated in this 4-people family, suggesting that this genetic mutation was the molecular etiology of the patient. We then used Sanger sequencing to verify the mutation status on 23 members of this family and found that all subjects had the same genotype-phenotype association, suggesting that this mutation might be the genetic factor leading to the corresponding eye abnormalities in this family. This study provided new evidence for the research in ASMD-related genes, suggesting that *PITX3*-related phenotypes may be caused by more complex mutation mechanisms. More in-depth research is needed to clarify the cause of this clinical variability.

## 5. Conclusion

The posterior pole cataract is characterized by the turbidity of the posterior pole (including lens cortex and lens capsule). Since the posterior pole is located in the optical center of the eye, it has a significant impact on vision. Although there have been studies on the changes in ultrastructure of the posterior capsular cataract, the exact pathogenesis mechanism is still unclear. So far, surgical treatment is the most effective method for treating congenital cataracts. However, since the clinical manifestations of congenital cataracts are complex and diverse and it is often associated with ocular structural abnormalities, the surgery is difficult to perform and there are frequent postoperative complications. Therefore, congenital cataracts are still a serious health problem causing considerable economic burden [31]. It is generally believed that the spatial distribution of opacity in the lens reflects the expression pattern of disease-causing genes during lens development, but the precise explanation for the specific hereditary cataract phenotype is still unknown. Here, we reported a large family in East China with different eye disease phenotypes caused by *PITX3* gene mutation. This study further proved the importance of combining clinical features with NGS to understand the biological basis of familial cataract phenotype caused by genetic mutations.

## Figures and Tables

**Table 1:** Clinical data of the 11 cataract patients.

	Items	II3	II5	II7	III3	III4	III6	III10	IV2	IV3	IV4	IV8
General information diagnosis	Gender	Male	Female	Female	Female	Female	Female	Female	Female	Male	Male	Male
	Age	53 years	66 years	64 years	23 years	39 years	30 years	31 years	14 years	7 years	4 years	3 months
	Congenital leukoplakia	Yes	Yes	Yes	Yes	No	No	No	No	Yes	No	Yes
	Congenital small cornea	Yes	Yes	Yes	Yes	No	No	No	No	Yes	No	Yes
	Congenital cataract	Yes	Yes	Yes	No	Yes	Yes	Yes	Yes	No	Yes	No

Surgical methods	Penetrating keratoplasty	Yes	No	No	No	No	No	No	No	Yes	No	Yes
	Cataract extraction	Yes	No	No	No	Yes	Yes	Yes	Yes	No	No	No
	Intraocular lens implantation	No	No	No	No	Yes	Yes	Yes	Yes	No	No	No

Co-occurrence	ASMD	Yes	Yes	Yes	Yes	No	No	No	No	Yes	Yes	Yes

**Table 2:** The known *PITX3* mutations associated with cataracts (NM_005029.4).

	Nucleic acid change	Protein change	Positions	Types	Mode	Numbers	Clinical phenotypes	References
1	c.38G > A	p.S13 N	Exon2	Missense	AD, heterozygous	2	ADCC, glaucoma, peters abnormality	[25, 30]
2	c.640_656dup	p.G220PfsX95	Exon4	Frameshift	AD, heterozygous	14	ADCC, PPC, ASMD	[14–17, 19, 23, 25, 30]
3	c.650delG	p.G217AfsX91	Exon4	Frameshift	AD/AR	2	PPC; microphthalmia with severe developmental delay (AR)	[14, 18]
4	c.542delC	p.P181LfsX127	Exon4	Frameshift	AD, heterozygous	1	PPC	[21]
5	c.640_656del	p.A214RfsX42	Exon4	Frameshift	AD/AR	2	PSC; microphthalmia with corneal sclerosis (AR)	[22, 26]
6	c.573delC	p.S192AfsX117	Exon4	Frameshift	AD, heterozygous	1	ADCC, ASMD	[23]
7	c.608delC	p.A203GfsX106	Exon4	Frameshift	AD, heterozygous	2	ADCC, PSC, nystagmus	[24, 26]
8	c.582delC	p.I194MfsX115	Exon4	Frameshift	AD, de novo	1	ADCC, microphthalmia, developmental delay, autism	[25]
9	c.669delC	p.L225WfsX84	Exon4	Frameshift	AD/AR	1	ADCC; ASMD, sclerosing cornea (AR)	[25]
10	c.797_814del	p.S266_A271del	Exon4	In-frame	AD, heterozygous	1	PSC	[27]
11	c.470−477dup	p.A160RfsX152	Exon4	Frameshift	AD, heterozygous	1	Nuclear cataract	[28]

*Note.* AD: autosomal dominant inheritance; AR: autosomal recessive inheritance; de novo: newly identified mutation; ADCC: autosomal dominant congenital cataract; ASMD: anterior segment mesenchyme dysplasia; PPC: posterior polar cataract; PSC: posterior subcapsular cataract.

## Data Availability

All the data generated or analyzed during this study are available from the corresponding author upon reasonable request.
